# Biochemistry and physiology of zebrafish photoreceptors

**DOI:** 10.1007/s00424-021-02528-z

**Published:** 2021-02-17

**Authors:** Jingjing Zang, Stephan C. F. Neuhauss

**Affiliations:** Department of Molecular Life Sciences, University of Zurich, Winterthurerstrase 190, CH – 8057 Zürich, Switzerland

**Keywords:** Zebrafish, Visual transduction, Photoreceptors, Cones

## Abstract

All vertebrates share a canonical retina with light-sensitive photoreceptors in the outer retina. These photoreceptors are of two kinds: rods and cones, adapted to low and bright light conditions, respectively. They both show a peculiar morphology, with long outer segments, comprised of ordered stacks of disc-shaped membranes. These discs host numerous proteins, many of which contribute to the visual transduction cascade. This pathway converts the light stimulus into a biological signal, ultimately modulating synaptic transmission. Recently, the zebrafish (*Danio rerio*) has gained popularity for studying the function of vertebrate photoreceptors. In this review, we introduce this model system and its contribution to our understanding of photoreception with a focus on the cone visual transduction cascade.

## Introduction

All vertebrates share a canonical retina with light-sensitive photoreceptors in the outer retina. These photoreceptors are of two kinds: rods and cones. Rod photoreceptors are characterized by higher light sensitivity and slower kinetics, mainly mediating monochromatic low-light vision [191, 50, 57, 105]. Cone photoreceptors on the other hand function under bright light, conveying luminance and color information. In vertebrates, they come in up to four different subtypes, depending on their peak absorption. Both photoreceptor types share a peculiar morphology with a large outer segment comprised of an ordered stack of discs, which contain the proteins of the visual transduction cascade. This biochemical pathway transforms the physical stimulus of light into a biological signal. Outer segments are modified primary cilia that are connected via an axoneme to the mitochondrium-rich inner segment [84]. Synapses of the photoreceptors are among the most complex synapses in the vertebrate brain, featuring ribbons that are thought to enable tonic glutamate release into the synapse [162, 175].

Photoreceptors have been intensively studied in different model organisms. Biochemists favor large bovine eyes for their large yield of proteins. Electrophysiologists favor the amphibians for their comparatively large photoreceptors and geneticists have mainly focused on rodent eyes due to the genetic amenities available in these systems.

More recently, the small tropical teleost zebrafish (*Danio rerio*) joined the ranks of model system for retinal research. Besides their favorable biological properties, such as small body size, easy maintenance, and large number of offspring, there are several properties of their visual system that have endeared this model system to visual scientists [157]. Unlike the rod-dominant amphibian or rodent retina, the majority of photoreceptors in zebrafish are cones, with about 92% in zebrafish larvae and about 60% in the adult [49, 2, 222]. The larval retina also serves as a model for the primate fovea, featuring a cone-rich acute zone responsible for prey detection [212]. Moreover, more than 70% of human genes have direct orthologues in the zebrafish genome [69], making zebrafish an ideal model to study eye or more specifically cone diseases in humans [61, 11, 113]. The genetic toolbox to manipulate zebrafish has massively expanded during the past decade, including DNA insertion, precisely controlled transgene expression, and CRISPR/Cas genome editing [131]. Because the zebrafish retina starts to transmit visual information at very early stages (3 days post fertilization (dpf)), the function of the visual system can be assessed at early larval stages. Finally, zebrafish larvae are transparent, making them well suited for live imaging (e.g., [135, 222]).

Zebrafish retina signaling with related ocular and retinal diseases have been reviewed recently [8, 123, 113, 126, 18, 11]. In this review, we will provide an overview of biochemical and physiological processes in zebrafish photoreceptors with a focus on the visual transduction cascade, the very first step of image-forming vision.

## Zebrafish outer retina

The zebrafish retina possesses one rod type and four morphologically and spectrally distinct cone subtypes, namely short single cones (ultraviolet (UV)-sensitive), long single cones (blue-sensitive), double cone accessory members (green-sensitive), and double-cone principle members (red-sensitive). Double cones exist in most vertebrates, but are absent in most placental mammals, elasmobranches, and catfish [44]. Zebrafish photoreceptors are coupled by gap junction, mainly mediated by Connexin 35 (the zebrafish homologue of mammalian Cx36) [111]. Fish photoreceptor coupling is regulated by the circadian clock, with cone-cone and rod-cone coupling being increased during nighttime [151, 111].

In the absence of pupillary reflexes, many lower vertebrates developed retinomotor movements to adapt to changes in light conditions. In darkness, a mobile part of photoreceptor inner segment, called the myoid, drives cones to elongate and rods to contract [124, 67]. Meanwhile, pigment granules (melanosomes) of the retinal pigment epithelium (RPE) concentrate at the basal part of the RPE. In this way, cone outer segments are buried deeply inside basal RPE while rod outer segments are optimally exposed to incoming light, by being situated far from pigment granules. During light adaptation, cones contract while rods elongate concomitant to pigment granule translocation towards the apical part of the RPE. Therefore, cone outer segments are exposed to light and the rod outer segments are protected by the RPE, akin to sunglasses [1]. The zebrafish retina shows adult-like retinomotor movement from 28 dpf on. Pigment granules take about an hour to migrate to fully light adapted position, while double cone outer segment contraction finishes in about 20 minutes [124, 67]

**Fig. 1:**
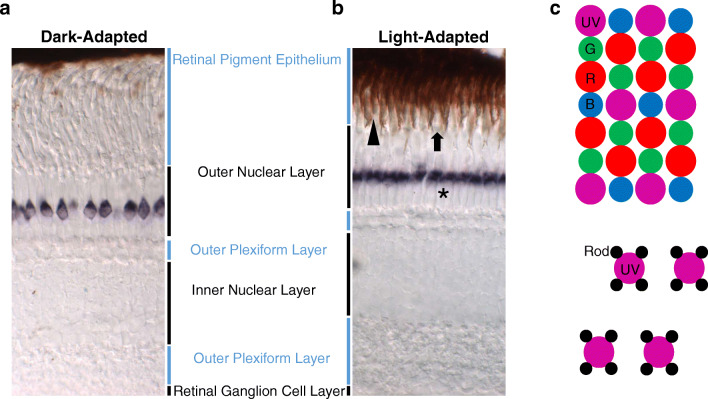
Adult zebrafish retina and photoreceptor mosaic. Dark-adapted adult zebrafish retina section (**a**) and light-adapted section (**b**) are organized into different cellular layers. The nuclei of rod and cone photoreceptors are located in the outer nuclear layer. During light adaptation, photoreceptor myoid drives cones to contract and rods to elongate to protect rods from over-bleaching, known as retinomotor movement. UV opsin (*sws1*) is labeled by in situ hybridization. Arrowhead denotes double cone. Arrow denotes blue cone. Star denotes cell body of rod. Schematic of the zebrafish photoreceptor planar mosaic arrangement (**c**) [153, 2, 49]. UV, UV cone; R, red cone; G, green cone; B, blue cone

Longitudinal sections of adult retina demonstrate that different photoreceptors are organized into different layers in zebrafish [153, 20] (Fig. 1a, b). The nuclei of rods are located distal to all cone nuclei. The nuclei of UV cones, blue cones, and double cones are located in the distal, medial, and proximal zones of the outer retina, respectively. Cone photoreceptors in the adult zebrafish retina are orderly arranged into a row mosaic pattern (Fig. 1c), in which a red cone neighbors a blue cone while a green cone neighbors a UV cone [2, 107]. Rods project into a square shape around each UV cone to form an integral photoreceptor mosaic [49].

The situation is different in the larval retina, where photoreceptors are anisotropically distributed [222]. All cone types are concentrated at the horizon and lower visual field, which may mediate color vision. UV cone density shows a peak at around 30° above the horizon, which is essential for visual prey hunting [212]. The upper visual field is dominated by rods, supporting the effectively achromatic vision towards the sky. This anisotropic distribution is adapted to the spectral content in the natural visual environment serving behavioral demands.

## Visual transduction cascade

**Fig. 2:**
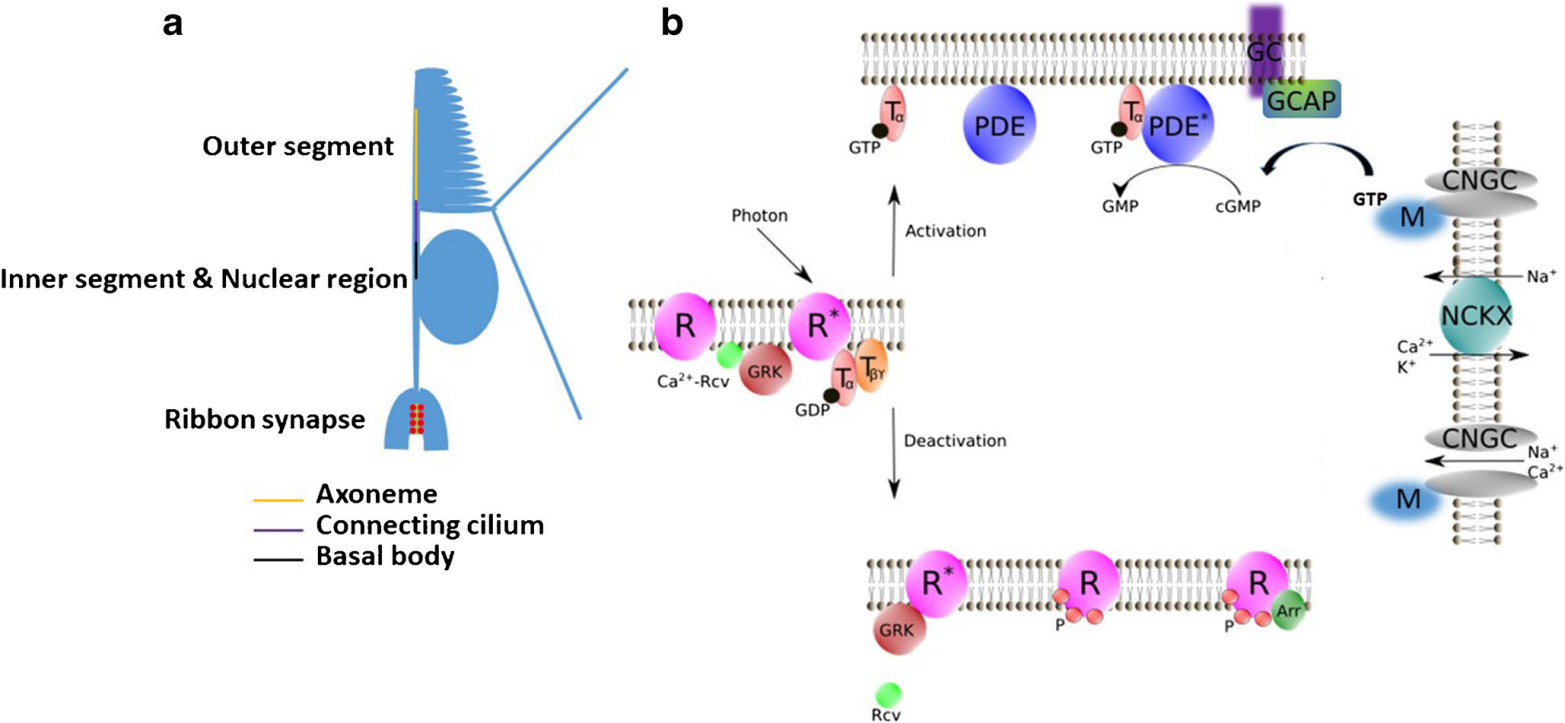
Cone photoreceptor morphology (**a**). Schematic representation of vertebrate visual transduction cascade and Ca^2+^-regulated deactivation processes (**b**). Photon absorption activates R. R* then triggers the exchange of GTP for GDP on the Tα. Tα-GTP binds to cyclic nucleotide PDE. Stimulated PDE hydrolyzes free cyclic guanosine monophosphate (cGMP). In darkness, CNGC allows an influx of Na^+^ and Ca^2+^, while during illumination CNGC is shut off by cGMP decrease. NCKX is not affected by light, which results in a light-induced intracellular Ca^2+^ concentration decline. Rcv modulates phosphorylation of R* via GRK in a Ca^2+^-dependent manner. Phosphorylated R then is fully deactivated by the binding of Arr. R, visual pigment (inactive); R*, light-activated visual pigment; Tα, transducin α subunit; Tβγ, transducin β and γ subunits; PDE, phosphodiesterase (inactive); PDE*, PDE-transducin α complex: NCKX, Na^+^/Ca^2+^, K^+^ exchanger; Arr, arrestin; GRK, G protein–coupled receptor kinase; Rcv, Recoverin; CNGC, cyclic nucleotide–gated ion channel; P, phosphorylation; M, CNG-modulin; GC, guanylate cyclase; GCAP, guanylate cyclase activating protein. Figure was drawn using Inkscape. Inkscape http://www.inkscape.org/. Reproduced with permission from Zang and Neuhauss [217]

The main function of photoreceptors is the capture of photons of visual light and the subsequent transformation of this physical stimulus into a biological signal, ultimately modifying the release of the neurotransmitter glutamate by the photoreceptor synapse [50, 105, 57, 22]. The visual transduction cascade and its regulation are among the best-understood trimeric G protein signaling pathways. All reactions take place in the outer segments of photoreceptors, most of them associated with the membrane (Fig. 2). Zebrafish genes involved in visual transduction cascade are summarized in Table 1.

**Table 1:** Summary of zebrafish genes involved in visual transduction cascade

Gene name	Protein encoded	Expression pattern in photoreceptor layer	Phenotype in zebrafish with abnormal gene expression	Mouse homologs	Associated human eye diseases
*opn1sw1*	UV opsin	UV cones		*Opn1sw*	Tritan color blindness [200, 201]
*opn1sw2*	Blue opsin	Blue cones			
*opn1mw1*	Green opsin	Green cones			Achromatopsia [130]
*opn1mw2*	Green opsin	Green cones			
*opn1mw3*	Green opsin	Green cones			
*opn1mw4*	Green opsin	Green cones			
*opn1lw1*	Red opsin	Red cones		*Opn1mw*	Achromatopsia [130]
*opn1lw2*	Red opsin	Red cones		*Opn1mw*	
*rho*	Rod opsin	Rods	Rod photoreceptor degeneration [218]	*Rho*	Night blindness [168], retinitis pigmentosa [43]
*gnat1*	Transducin α subunits	Rods and UV cones		*Gnat1*	Night blindness [128]
*gnb1a*	Transducin β subunits	Rods and UV cones		*Gnb1*	
*gnb1b*	Transducin β subunits	Rods and UV cones		*Gnb1*	
*gngt1*	Transducin γ subunits	Rods and UV cones		*Gngt1*	
*gnat2*	Transducin α subunits	Cones	Largely reduced photoresponse [21]	*Gnat2*	Achromatopsia [91]
*gnb3a*	Transducin β subunits	Cones		*Gnb3*	
*gnb3b*	Transducin β subunits	Cones		*Gnb3*	
*gngt2a*	Transducin γ subunits	Cones		*Gngt2*	
*gngt2b*	Transducin γ subunits	Cones		*Gngt2*	
*pde6a*	PDE catalytic α subunit	Rods and UV cones		*Pde6a*	Autosomal recessive retinitis pigmentosa [72]
*pde6b*	PDE catalytic β subunit	Rods and UV cones		*Pde6b*	Autosomal recessive retinitis pigmentosa [122]
*pde6ga*	PDE inhibitory γ subunit	Rods and UV cones		*Pde6g*	
*pde6gb*	PDE inhibitory γ subunit	Rods and UV cones		*Pde6g*	
*pde6c*	PDE catalytic α′ subunit	Cones	Diminished cone ERG and OKR, and cone degeneration [134, 172]	*Pde6c*	Cone dysfunction and achromatopsia [27, 59, 186]
*pde6ha*	PDE inhibitory γ′ subunit	Cones		*Pde6h*	
*pde6hb*	PDE inhibitory γ′ subunit	Cones		*Pde6h*	
*pde6i*	PDE inhibitory γ′ subunit				
*cnga1a*	CNG channel α1 subunit	Rods		*Cnga1*	Autosomal recessive retinitis pigmentosa [42]
*cnga1b*	CNG channel α1 subunit	Rods		*Cnga1*	
*cngb1a*	CNG channel β1 subunit	Rods		*Cngb1*	Autosomal recessive retinitis pigmentosa [5, 14, 93]
*cngb1b*	CNG channel β1 subunit	Rods		*Cngb1*	
*cnga3a*	CNG channel α3 subunit	Cones		*Cnga3*	
*cnga3b*	CNG channel α3 subunit	Cones		*Cnga3*	
*cngb3.1*	CNG channel β3 subunit	Cones		*Cngb3*	
*cngb3.2*	CNG channel β3 subunit	Cones		*Cngb3*	
*grk1a*	G protein–coupled receptor kinase 1a	Rods	Overexpression of *grk1a* in rods shows minor effect [194]	*Grk1*	Oguchi disease [209]
*grk1b*	G protein–coupled receptor kinase 1b	Cones	Delayed ERG response recovery and reduced temporal contrast sensitivity[31]	*Grk1*	
*grk7a*	G protein–coupled receptor kinase 7a	Cones	*grk7a* knockdown [152], *grk7a* knockout [31], ectopic expression of *grk7a* in rods [194]		
*grk7b*	G protein–coupled receptor kinase 7b	UV cones			
*arrSa*	ArrestinSa	Rods and UV cones		*Arr1*	Oguchi disease [58]
*arrSb*	ArrestinSb	Rods and UV cones		*Arr1*	
*arr3a*	Arrestin3a	Double cones	Delayed ERG response recovery and decreased temporal contrast sensitivity [148]	*Arr3*	
*arr3b*	Arrestin3b	Blue and UV cones		*Arr3*	
*rgs9a*	Regulators of G protein signaling 9a	Cones		*Rgs9*	Bradyoposia [133]
*rgs9b*	Regulators of G protein signaling 9b	Rods		*Rgs9*	
*gucy2e*	Guanylate cyclase E	Rods and UV cones	Outer segment loss and shortening, OMR defects [176]	*Gucy2e*	Leber congenital amaurosis 1 [142]
*gucy2f*	Guanylate cyclase F	Rods and UV cones		*Gucy2f*	
*gucy2d*	Guanylate cyclase D	Cones	OKR and OMR impairments [127], PDE6c downregulation [79]	*Gucy2d*	
*slc24a1*	Na^+^/Ca2^+^, K^+^ exchanger 1			*Slc24a1*	Congenital stationary night blindness [150]
*slc24a2*	Na^+^/Ca2^+^, K^+^ exchanger 2			*Slc24a2*	
*rcv1a*	Recoverin 1a	Rods and UV cones	Accelerates photoresponse recovery [215]	*Rcv1*	
*rcv1b*	Recoverin 1b	Cones			
*rcv2a*	Recoverin 2a	Cones	Accelerates photoresponse recovery [215]		
*rcv2b*	Recoverin 2b	Cones	Accelerates photoresponse recovery [215]		
*gcap1*	Guanylate cyclase activation protein 1	Rods and UV cones		*Guca1a*	Autosomal dominant cone dystrophy [140]
*gcap2*	Guanylate cyclase activation protein 2	Rods and UV cones		*Guca2b*	Autosomal dominant retinal dystrophies [160]
*gcap3*	Guanylate cyclase activation protein 3	Cones	Prolonged photoresponse recovery [9]		
*gcap4*	Guanylate cyclase activation protein 4	Cones			
*gcap5*	Guanylate cyclase activation protein 5	Cones			
*gcap7*	Guanylate cyclase activation protein 7	Cones			
*eml1*	CNG-modulin	Cones	Reduced light sensitivity [94]	*Eml1*	

The photoreceptor outer segment is a cylindrical structure comprised of an ordered stack of disc-shaped membranes, allowing a high concentration of transmembrane visual pigments and increasing the probability of photon catch [92].

Rod and cone photoreceptors share a generally similar visual transduction cascade, but adopt rod- or cone-specific protein isoforms for many of the cascade’s components. The evolution of these photoreceptor-specific paralogues is a well-studied paradigm for the fate of duplicated genes in evolution [103]. This is particularly true for teleost genomes that underwent a lineage-specific whole-genome duplication, following two rounds of whole-genome duplications in the early vertebrate lineage [7, 190, 184].

The generation, deletion, and fate of these duplicated genes add a fascinating complexity to the teleost visual transduction cascade that is beyond the scope of this review. However, the multitude of gene variants to be discussed in the following is the direct consequence of whole-genome duplications in the past [63, 103, 104, 106, 99, 100, 60].

The visual transduction cascade is initiated by the absorption of photons by opsins. These G protein–coupled 7-transmembrane receptors are covalently bound to a light-sensitive chromophore via a Schiff base forming the photopigment complex [73]. Upon the absorption of a photon, chromophore (most commonly vitamin A_1_ 11-*cis*-retinal) in the photopigment complex isomerizes to *all*-*trans*-retinal, which activates the opsin (now referred to R*) by inducing a transformational change [51, 52]. Zebrafish cones express a total of 8 cone opsins, namely *opn1sw1* (also known as *sws1*), *opn1sw2* (also known as *sws2*), *opn1mw1* (also known as *rh2-1*), *opn1mw2* (also known as *rh2-2*), *opn1mw3* (also known as *rh2-3*), *opn1mw4* (also known as *rh2-4*), *opn1lw1* (also known as *lws1*), and *opn1lw2* (also known as *lws2*) [2, 145, 182]. Hence, there are four green (short wave length) and two red (long wavelength) opsin variants. These variants have slightly different peak absorption properties potentially allowing a bewildering range of fine-tuning of color perception [30, 23]. The expressions of these multiple *rh2* and *lws* genes follow a spatiotemporal order during development [182]. Rod photoreceptors express only a single-rod opsin gene *rho* (also known as *rh1*) [218]. Mutations in human rod opsin may produce night blindness or retinal degeneration, while cone opsin defects may lead to achromatopsia [168, 43, 130, 200, 201]. For years, vitamin A_1_-based photopigment has been recognized as the sole photopigment existing in zebrafish photoreceptors under standard laboratory conditions [23, 30]. The peak absorption spectra (*λ*_max_) of A_1_-based photopigments differ markedly and cover a wide spectrum from 355 nm (UV) to 558 nm (red) in vivo. However, thyroid hormone (TH) treatment or colder water temperature may result in a transition from A_1_ to vitamin A_2_ (11-*cis* 3,4-didehydroretinal)-based photopigments. This demonstrates a functional A_1_-A_2_ photopigment interchange system in zebrafish [159, 3, 48]. The *λ*_max_ of A_2_-based photopigments shifts towards longer wavelength relative to A_1_-based photopigment [115, 66]. This interchange system is frequently observed in freshwater fishes and amphibians, and may be adapted to the red-shifted light environment in fresh water compared with marine and terrestrial environments [149, 197, 222]. Another mechanism to tune photopigments is to change opsin expression levels. TH treatment has been reported to reduce *lws2* (548 nm) and *rh2-1* (467 nm), while increasing *lws1* (558 nm) and *rh2-2* (488 nm) in larvae, favoring the opsins with longer *λ*_max_ [119]. Both the mechanisms red-shift the zebrafish photoreceptor spectral sensitivity. Moreover, in TH receptor-defective fish, retinal progenitors designed to become red cones are transfated into UV cones, providing another mechanism for TH to regulate long-wavelength vision [180, 195, 37].

Besides the visual opsins, the zebrafish genome harbors 32 nonvisual opsin genes, which encode opsins forming functional photopigments with different chromophores [35, 34, 56]. Many, but not all of them, are expressed in the photoreceptor layer. Their functions in photoreceptors are largely unknown, but a role in circadian light entrainment is discussed [56, 174, 26].

Activated opsin (R*) interacts with the trimeric G protein transducin [22, 50, 105]. Binding of R* to transducin results in the replacement of GDP by GTP at the active site of the transducin α subunit. This nucleotide exchange dissociates the activated α subunit (Gα*) and the heterodimer of β and γ subunits (Gβγ). Gα* then binds to the cGMP Phosphodiesterase 6 (PDE6) [91, 128].

Zebrafish rod and cone photoreceptors express different variants of three subunits [99, 28]. In rods, *gnat1* encodes transducin α subunits, *gnb1a* and *gnb1b* encode β subunits, and *gngt1* encodes γ subunits (all these variants possibly also in UV cones), while in cones, *gnat2* encodes α subunits, *gnb3a* and *gnb3b* encode β subunits, and *gngt2a* and g*ngt2b* encode γ subunits.

Surprisingly, a zebrafish mutant defective in the cone-specific *gnat2* gene (*no optokinetic response f* (*nof*)) shows a residual photoresponse that needs to be mediated by an unknown transducin-independent mechanism [21].

Interestingly, both Gα and Gβ show massive light-induced translocation from rod outer segment to inner segment in mice, which may contribute to light adaptation in rods [170]. However, Gα translocation has not been observed in zebrafish cones (or mouse cone), indicating light adaptation mechanisms may vary between rods and cones [85, 46, 114].

When Gα* binds to PDE6, two PDE6 inhibitory subunits dissociate from the active sites and allow the activation of PDE6 to hydrolyze cGMP [32]. The rod PDE6 variant is expressed as a heterotetramer consisting of two catalytic α and β subunits encoded by *pde6a* and *pde6b*, and two identical inhibitory γ subunits encoded by *pde6g*. Cone PDE6 comprises two homodimers of two catalytic α′ subunits encoded by *pde6c* and two inhibitory γ′ subunits encoded by *pde6h* [106, 62, 32, 72, 122].

Zebrafish retain the same set of catalytic subunit genes as in humans (*pde6a*, *pde6b*, and *pde6c*), while inhibitory subunits are encoded by duplicated paralogues: *pde6ga* and *pde6gb* in rods and possibly UV cones and *pde6ha* and *pde6hb* in all cones [100, 134]. An additional inhibitory subunit gene *pde6i* has also been found in zebrafish, and some other lower vertebrates including fish (teleost and non-teleost) and amphibians [100].

Mutations in the cone-specific *pde6c* gene are associated with cone dysfunction in human patients with achromatopsia [27, 59, 186]. Mutations in cone-catalytic subunit *pde6c* result in almost diminished cone electroretinogram (ERG) and optokinetic response (OKR), and cone photoreceptor degeneration in zebrafish [134, 172]. The mechanism underlying cone degeneration is unknown and is not linked to increased cytosolic Ca^2+^ levels [118].

Ultimately, the visual transduction cascade regulates the opening of cyclic nucleotide-gated (CNG) ion channels. These non-selective cation channels are opened by cGMP binding [210]. Falling cGMP concentration due to cGMP hydrolysis by PDE6 leads to the closure of these CNG channels, suppressing the circulating dark current and resulting in photoreceptor hyperpolarization. CNG channels are heteromeric proteins consisting of α and β subunits [81, 125]. Rod channels are assembled from 3 CNGA1 subunits and 1 CNGB1, while cone channels are assembled from 2 CNGA3 subunits and 2 CNGB3 subunits [202, 220, 221, 102].

Mutations in CNGA1 and CNGB1 have been identified in human patients with autosomal-recessive retinitis pigmentosa [14, 5, 93, 42]. In zebrafish, all visual CNG channel genes have retained two paralogues, but no additional information is available.

## Regulation of visual transduction

At the biochemical level, visual transduction is mainly regulated by its deactivation kinetics. To deactivate the visual transduction cascade, deactivation of both R* and Gα-PDE* complex and the restoration of cGMP concentrations are required [22, 50].

The lifetime of R* is tightly regulated by arrestin proteins that efficiently inactivate photopigmet by binding to its phosphorylated form. Therefore, the first step of R* inactivation is phosphorylation. R* is phosphorylated by G protein–coupled receptor kinases (GRKs). Mice and rats express only GRK1 in both rods and cones, while humans express GRK1 in rods and GRK1 and GRK7 in cones [219, 117, 165, 199]. In zebrafish, both visual *grk* genes are present as two paralogues. *grk1a* is expressed exclusively in rods, *grk1b* and *grk7a* in all cones, and *grk7b* only in UV cones [152, 196] (unpublished data). GRK deficiency in humans leads to Oguchi disease, which is characterized by a delay of rod recovery [209]. A *grk7a* knockdown model produces largely delayed ERG response recovery and reduced temporal contrast sensitivity in the OKR [152]. Another study demonstrates similar but more modest effects in either *grk1b* or *grk7a* mutants [31].

Overexpression of *grk1a* in zebrafish rods shows minor effect on rod photoresponse, suggesting that endogenous GRK1a protein is already at saturation levels. Ectopic expression of cone *grk7a* in rods resulted in cone-like rod responses [194].

The binding of arrestin completely deactivates the phosphorylated photopigment [98, 203]. In the mouse retina, both rod (ARR1) and cone (ARR3) arrestins are co-expressed in cone photoreceptors [132, 203]. Mutations in ARR1 are a cause of Oguchi disease in human [58]. In zebrafish, *arrsa* and *arrsb* (orthologues of *Arr1*) are expressed in rods while *arr3a* exists in double cones and *arr3b* exists in blue and UV cones, indicating subfunctionalization of the two paralogues. *arr3a* knockdown resulted in a severe delay in ERG response recovery and decreased temporal contrast sensitivity [148].

Regulators of G protein signaling 9 (RGS9) act as GTPase activating protein to deactivate Gα*-PDE complex [17]. Mammals have a single *Rgs9* gene, while zebrafish have two *rgs9* genes, with *rgs9a* being expressed in cones and *rgs9b* in rods [33, 104] (unpublished data). Inactivating mutations in humans lead to bradyopsia, a rare condition characterized by slower photoreceptor deactivation [133]. A landmark study using Rgs9 overexpression in mice demonstrated its crucial role to rate-limit rod visual transduction recovery [96].

To restore the dark current, cGMP needs to be resynthesized by membrane-bound guanylate cyclases (GCs) [88, 167]. Photoreceptor-specific GCs are regulated by the small Ca^2+^-binding guanylate cyclase activation proteins (CGAPs) [90].

Mammals have two photoreceptor-specific GCs, GC-E (known as GC1) and GC-F (known as GC2), both of which are co-expressed in rods and cones [103, 88, 60]. GC-E is more concentrated in cones, while the expression of GC-F is more prominent in rods. Mutations in GC-E have been shown to cause Leber congenital amaurosis 1 (LCA1), a severe form of pediatric blindness in humans [142]. The zebrafish possess 3 GCs. GC-E (known as GC1), GC-F (known as GC2), and GC-D (known as GC3) are encoded by *gucy2e* (previous name *gucy2f*), *gucy2f* (previous name *gc2*), and *gucy2d* (previous name *gc3*), respectively. Both *gucy2e* and *gucy2f* are expressed in rods and UV cones, while *gucy2d* encodes the only cone-specific GC in all cone subtypes [55, 144].

A zebrafish *gucy2d* mutant has been identified in behavioral screen by displaying OKR and optomotor response (OMR) impairments [127]. PDE6c protein levels are downregulated in *gucy2d* knockdown larvae, indicating the interdependence between these two regulators of cGMP metabolism [79]. A knockdown of the *gucy2d* gene results in the loss and shortening of outer segments and defects in the OMR [176].

In darkness, the open non-selective CNG channels mediate a Ca^2+^ influx into the photoreceptor outer segment. Ca^2+^ efflux via Na^+^/Ca^2+^, K^+^ exchanger (NCKX) balances this influx, producing a moderately high intracellular Ca^2+^ concentration as shown in rods of different species [101, 207]. Under light illumination, CNG channels are closed due to the decrease in cGMP concentration, while Ca^2+^ efflux continues, resulting in a decrease of intracellular Ca^2+^ concentration in the outer segment [211]. This light-induced Ca^2+^ decline can be simultaneously measured with light response in zebrafish UV cones, demonstrating similar kinetics of Ca^2+^ extrusion via NCKX to that of CNG channel current [109].

NCKX proteins are encoded by *SLC24* gene family members. They show a cell-type-specific expression with NCKX1 being expressed in rods and NCKX2 in cones [193, 147, 143, 150]. NCKX2-deficient mice show no or only mild functional defect, suggesting that compensating transporters may mediate ion exchange as well [112, 156]. A recent study proposed that NCKX2 and NCKX4 cooperated to facilitate the rapid and efficient extrusion of Ca^2+^ from mouse cones. NCKX4 has its well-established function in olfactory sensory neurons and is similarly expressed in all cones in the zebrafish retina [192]. The expression pattern of the other NCKX coding genes is unknown in zebrafish, but studies in the striped bass show expression of *nckx1* in rods and four splice variants of *nckx2* in cones [137].

The reduction of cytoplasmic Ca^2+^ negatively feedbacks to the phototransduction cascade, triggering the rapid photoresponse recovery and facilitating photoreceptor adaptation to background light [120, 129]. During light adaptation, photoreceptor light sensitivity is reduced and response kinetics is accelerated, to avoid saturation and to operate across a wide range of environmental light intensity [50]. This has been achieved by mechanisms that primarily involve the regulation of GRKs by Recoverin, GCs by GCAPs, and CNG channels by CNG-modulin (or Calmodulin) [138, 191].

Recoverin (RCV) is a small neuronal calcium sensor (NCS), which is primarily located in vertebrate photoreceptors. Upon Ca^2+^ binding, RCV undergoes a pronounced conformational change, the so-called Ca^2+^-myristoyl switch, which translocates the proteins from a cytosolic form to a membrane tethered conformation, allowing targeting and inhibiting GRK proteins [82, 166, 183, 6, 40, 83, 217]. Light stimulation reduces intracellular Ca^2+^ concentration, allowing the Ca^2+^-free RCV releasing GRK. GRK disinhibition accelerates R* phosphorylation, enabling arrestin binding.

While there is only one RCV isoform in mammals (RCV1), four *rcv* genes are encoded in the zebrafish genome (*rcv1a*, *rcv1b*, *rcv2a*, and *rcv2b*) [215]. *rcv1b*, *rcv2a*, and *rcv2b* are cone RCV, while *rcv1a* is expressed in rods and UV cones. Mouse RCV1 experiences a remarkable light-induced translocation from outer and inner segment towards synaptic terminals in rods, which has not been observed in zebrafish photoreceptors by studying all zebrafish RCVs [177] (unpublished observation). Downregulation of cone RCV accelerates photoresponse recovery, but this effect is abolished when cone GRK7a is simultaneously knocked-down. This result not only indicates that RCV regulates opsin deactivation via GRK, but also demonstrates that the cone opsin deactivation kinetics dominates the overall photoresponse shut off kinetics in vivo [215]. Interestingly, different RCVs contribute at distinct light intensities. This implies different Ca^2+^ sensitivities for these RCVs, since intracellular Ca^2+^ concentration correlates with light levels [158]. Indeed, a recent biochemical work demonstrated distinct Ca^2+^ affinities, Ca^2+^-dependent membrane binding, and Ca^2+^-induced conformational changes among zebrafish isoforms [45]. Furthermore, salamander cone photoresponse, but not rod response, is also dominated by a Ca^2+^-sensitive mechanism [121, 216]. If the Ca^2+^-sensitive dominance is a general feature in cone photoresponse, it may contribute to the more powerful light adaptation of cones compared to rods.

To restore the dark current, cGMP needs to be resynthesized by GC, which is under the regulation of small Ca^2+^-binding proteins called GCAPs [90, 39]. GCAPs belong to the superfamily of EF-hand Ca^2+^-binding proteins, harboring four EF-hand Ca^2+^-binding motifs, three of which are functional [89]. Unlike RCVs, GCAPs do not undergo a classical Ca^2+^-myristoyl switch, but the myristoyl group does play an important role to regulate GCAP properties, including Ca^2+^ sensitivity, GC affinity, and the catalytic efficiency of the enzyme. Ca^2+^-binding GCAPs together with GCs form GC/GCAP complex in darkness. Ca^2+^ reduction during light exposure triggers a conformational change in GCAPs, which results in a transformational change within the GC/GCAP complex, increases GC catalytic activity and reopens the CNG channels. During light adaptation, the Ca^2+^-sensitive GCAP activity will also prevent the closure of all CNG channels and keep photoreceptors responsive.

GCAP1 and 2 are expressed in mammalian rods and cones. The human (but not the mouse) genome also processes a cone-specific CGAP3 [75, 140, 160]. Zebrafish photoreceptors express six GCAPs, of which *gcap3*, *4*, *5*, and *7* are restricted to cones and *gcap1* and *2* are exclusively expressed in rods and UV cones [76, 144, 54]. These isoforms show distinct Ca^2+^ sensitivities of GC activation, Ca^2+^/Mg^2+^-dependent conformational changes, and Ca^2+^-binding affinities [164, 179]. Light exposure allows intracellular Ca^2+^ fluctuating to different levels, in which distinct CGAPs may reach their optimal working range.

GCAP3 is first expressed in a non-myristoylated form in larvae and then becomes myristoylated in the adult retina [54]. Although GCAP3 has been shown to produce the highest Ca^2+^-dependent activation of GCs in native zebrafish retina, *gcap3* knockdown does not induce any visual behavioral abnormalities [55]. In another study, GCAP3 in green cone was inactivated by antibody injections. Whole-cell patch clamp recordings demonstrated that the photoresponse recovery is strongly prolonged, confirming GCAP3 function to activate GC to restore CNG channel current in cones [9].

cGMP affinity of CNG channels is regulated in a Ca^2+^-dependent manner in all sensory neurons [19]. Ca^2+^ cannot directly bind to the channels but work via modulator proteins, which have been identified as calmodulin in mammalian rods and CNG-modulin in fish cones [70, 146]. However, the contribution of CNG channel modulation by Ca^2+^ in regulating light adaptation is very limited in rods [29, 95]. On the other hand, CNG-modulin has been shown to regulate the cGMP dependence of CNG channels in a Ca^2+^-sensitive manner, and to modulate the light response kinetics in striped bass cone [146]. CNG-modulin is encoded by the *eml1* gene in zebrafish. *eml1* knockdown reduces the light sensitivity of dark-adapted and light-adapted cones; the sensitivity cannot be restored to wild-type levels [94]. These experiments demonstrate a stronger Ca^2+^ feedback to CNG channels in cones compared to rods.

## Outer segment: a specialized primary cilium

Photoreceptor outer segments are strongly modified specialized primary cilia, sharing many general structural and biochemical features of cilia [77]. Outer segment stacked discs are arranged on the side of a microtubule-based axoneme, anchoring inside the inner segment through a connecting cilium and its basal body. Therefore, the connecting cilium, known as the transition zone in other cell types, connects outer and inner segment, mediating bi-directional protein trafficking [181]. Dysfunctions of primary cilia result in human disorders referred to as ciliopathies, which were reviewed elsewhere [11].

Outer segments are constantly bombarded by photons and their integrity is endangered by radical oxygen species. Since photoreceptors, like most neurons of the central nervous system, cannot be replaced, photoreceptors constantly rejuvenate themselves by renewing their outer segments. New discs are synthesized by ciliary membrane evagination at the base of the outer segment as the ciliary ectosomes, which then is elongated, flattened, and enclosed inside the outer segment [87, 173, 38, 171]. The tips of the outer segments, containing the oldest and potentially damaged membranes, are phagocytosed and digested by RPE cells. Although outer segment renewal/shedding is essential for photoreceptor homeostasis and survival, molecular mechanisms underlying its regulation are still poorly understood.

Recent works on zebrafish have contributed significantly to our understanding of the molecular mechanisms behind photoreceptor outer segment shedding and renewal. The zebrafish lends itself ideally to transgenically label cellular structures or cells, as Willoughby and colleagues have used elegantly for the outer segment [205]. They devised a stable line with heat shock–inducible fluorescent membrane protein that allowed them to follow the renewal and shedding of the rod outer segments as an updated experimental approach to the classic radioactive labeling method [214]. This line was then used in a high-content small-molecule screens that among others identified an involvement of cyclooxygenase in outer segment growth, gamma secretase in outer segment shedding, and mTOR in RPE phagocytosis [25].

Some earlier studies demonstrated that disc shedding in frog and cat was initiated by light [15, 53]. A recent zebrafish study using PDE6 inhibitors to block the visual transduction cascade mimicking constant dark conditions indeed inhibited rod outer segment shedding [24]. Interestingly, mammalian rod outer segment shedding remains in constant darkness, instead showing circadian clock controlling disc shedding mechanism [108, 185, 64, 74].

Given the nature of the outer segment, it comes as no surprise that many genes associated with intracellular and ciliary trafficking are involved in outer segment generation and maintenance.

The most abundant protein that needs to be shipped out to the outer segment is rhodopsin. Every second, around 70 rhodopsin molecules are trafficked from the inner to the outer segment [213, 204, 141]. Detailed studies of rhodopsin transport in frogs showed that RAB8, a small GTPase, coats rhodopsin-carrier vesicles and directs them to a selective barrier at the base of connecting cilium [139, 36]. In live imaging experiments in zebrafish, RAB8-directed rhodopsin trafficking in rods has been directly visualized in vivo [135]. The correct localization of RAB8 at the base of the outer segment is regulated by components of the connecting cilium itself, such as CC2D2A and further interaction partners, such as Ninl and MICAL3 [10, 12].

About 10% of outer segment is renewed every day in mammalian photoreceptors [108]. Therefore, intraflagellar transport (IFT), which contributes primarily to traffic visual transduction proteins into the outer segment, is important for outer segment development and structure [77]. IFT-B complex and kinesin motors mediate anterograde movement towards the distal outer segment, while IFT-A and dynein motors mediate retrograde movement towards the cell body [154].

A series of zebrafish studies contributed greatly to our understanding of the mechanism underlying IFT. Mutations affecting the IFT-B complex (IFT52, IFT57, IFT88, IFT172) lead to defects in outer segment formation and/or maintenance, finally resulting in both rod and cone degeneration [188, 41, 65]. Biochemical assays indicated that IFT20, a IFT-A member, requires IFT57 to associate with the IFT particle [97]. In another study, TNF receptor-associated factor 3 interacting protein 1 (TRAF3IP1) was shown to bind to IFT20. It can also interact with RAB8 via Rabaptin5, an endocytosis regulator. This demonstrates a connection between the IFT particle and the GTPase pathway, known to facilitate protein complex assembling [136].

Moreover, microtubular motors play an essential role in transporting IFT complexes. KIF17, kinesin-2 family member, is involved in ciliogenesis [206]. It is located all over zebrafish cones but concentrates at the basal body and the distal tip of the axoneme [13]. Knockdown of *kif17* disrupts outer segment structure and mislocates visual transduction proteins [78]. Disc shedding is also promoted by KIF17 and eliminated in its absence [110].

## Ribbon synapses

Non-spiking photoreceptors respond and adapt to a wide range of light intensities. The light-induced CNG channel closure generates the graded changes in membrane potential, which in turn regulates tonic neurotransmitter glutamate release at the presynaptic terminals [175, 163, 187]. This graded signaling is facilitated by specialized ribbon synapses, which hold a dense array of synaptic vesicles near active zones along their surface and were firstly identified by electron microscopy as electron dense structures in guinea pig rod synapses [169].

Work on zebrafish has helped to identify the key components of ribbon synapses and their function in signal transmission.

Ribeye is the most abundant protein in the synaptic ribbon [163]. In the zebrafish retina, both *ribeyea* and *ribeyeb* are present in the photoreceptors while *ribeyea* also shows expression in bipolar cells. Downregulation of *ribeyea* diminishes OKR and reduces ribbon length and number [198, 116].

Synaptojanin (Synj1) is a polyphosphoinositide phosphatase regulating clathrin-mediated endocytosis in conventional synapses [155]. A zebrafish *synj1* null mutation (*nrc*) shows unanchored “floating” ribbons and reduced synaptic vesicles in cone but not rod synapses[189, 68], associated with defect in vision [4].

Photoreceptor L-type voltage-dependent calcium channels (Ca_v_1.4) are located in the vicinity of synaptic ribbons and mediate exocytosis [187]. In darkness, they are opened by the depolarized photoreceptor membrane potential, resulting in calcium-dependent glutamate release. Ca_v_1.4 are heteromultimeric protein complexes comprising of a pore-forming α_1_F subunit, encoded by *CACNA1F*, and accessory β and α_2_δ subunits, encoded by *CACNB2* and *CACNA2D4*, respectively. Mutations in *CACNA1F* gene result in X-linked congenital stationary night blindness type 2 and cone-rod dystrophy in human [16, 178]. Two paralogues, *cacna1fa* and *cacna1fb* are identified in zebrafish with *cacna1fa* being expressed in photoreceptors while *cacna1fb* only existing in the inner retina [80]. CACNA1Fa protein exclusively accumulates at the outer plexiform layer and its null mutants (*wud*) present thinner outer plexiform layer, defective ERG, completely absent of synaptic ribbons, and mislocalized Ribeyeb.

Mutations in human *CACNA2D4* are related to autosomal recessive cone dystrophy, while rods in different *CACNA2D4* knockout mouse lines are even more severely affected, showing missing or largely defective scotopic and photopic ERG response [208, 86, 71]. More recently, another study focused on zebrafish cacna2d4 encoding Cav1.4 α2δ subunit [116]. *cacna2d4* is duplicated in zebrafish as *cacna2d4a* and *cacna2d4b*. Double KO shows reduced pore-forming CACNA1Fa expression and minor defects in both visual function and ribbon structure. The zebrafish KO model is associated with similar moderate phenotype in human patients, providing a comprehensive tool to study the related human eye disorders.

Zebrafish show a peculiar phenomenon of disassembled ribbon synapses at least in the larval retina during the night. At light onset, the presynaptic structure is rapidly reassembled for function [47]. This unusual mechanism may have evolved to save energy in rapidly growing larvae.

## Conclusion

The zebrafish retina serves as an important model of cone photoreceptor and has already contributed significantly to our understanding of photoreceptor maintenance and function. With its ever-increasing toolbox of imaging and genetic techniques, it will continue to crucially help us further in investigating the outer retina and its diseases.
